# Early Proteomic Characteristics and Changes in the Optic Nerve Head, Optic Nerve, and Retina in a Rat Model of Ocular Hypertension

**DOI:** 10.1016/j.mcpro.2023.100654

**Published:** 2023-10-02

**Authors:** Danting Lin, Shen Wu, Ying Cheng, Xuejing Yan, Qian Liu, Tianmin Ren, Jingxue Zhang, Ningli Wang

**Affiliations:** 1 Beijing Institute of Ophthalmology, Beijing Tongren Eye Center, Beijing Tongren Hospital, Capital Medical University, Beijing Ophthalmology & Visual Sciences Key Laboratory, Beijing, China; 2 Beijing Institute of Brain Disorders, Collaborative Innovation Center for Brain Disorders, Capital Medical University, Beijing, China; 3 Beijing Key Laboratory of Fundamental Research on Biomechanics in Clinical Application, Capital Medical University, Beijing, China

**Keywords:** glaucoma, proteomics, retina, optic nerve head, optic nerve

## Abstract

The pathogenesis of glaucoma is still unknown. There are few studies on the dynamic change of tissue-specific and time-specific molecular pathophysiology caused by ocular hypertension (OHT). This study aimed to identify the early proteomic alterations in the retina, optic nerve head (ONH), and optic nerve (ON). After establishing a rat model of OHT, we harvested the tissues from control and glaucomatous eyes and analyzed the changes in protein expression using a multiplexed quantitative proteomics approach (TMT-MS3). Our study identified 6403 proteins after 1-day OHT and 4399 proteins after 7-days OHT in the retina, 5493 proteins after 1-day OHT and 4544 proteins after 7-days OHT in ONH, and 5455 proteins after 1-day OHT and 3835 proteins after 7-days OHT in the ON. Of these, 560 and 489 differential proteins were identified on day 1 and 7 after OHT in the retina, 428 and 761 differential proteins were identified on day 1 and 7 after OHT in the ONH, and 257 and 205 differential proteins on days 1 and 7 after OHT in the ON. Computational analysis on day 1 and 7 of OHT revealed that alpha-2 macroglobulin was upregulated across two time points and three tissues stably. The differentially expressed proteins between day 1 and 7 after OHT in the retina, ONH, and ON were associated with glutathione metabolism, mitochondrial dysfunction/oxidative phosphorylation, oxidative stress, microtubule, and crystallin. And the most significant change in retina are crystallins. We validated this proteomic result with the Western blot of crystallin proteins and found that upregulated on day 1 but recovered on day 7 after OHT, which are promising as therapeutic targets. These findings provide insights into the time- and region-order mechanisms that are specifically affected in the retina, ONH, and ON in response to elevated IOP during the early stages.

## Highlights

• Early proteomic alterations in glaucomatous retina, optic nerve head and optic nerve.
• Ocular hypertension caused mitochondrial dysfunction in all three tissues.
• The A2m protein was upregulated across two time points and three tissues stably.
• The most significant change between the two time points in retina are crystallins.
• The mainly change between the two time points in ONH are ECM/integrin pathways.

## Graphical Abstract

**Figure:**
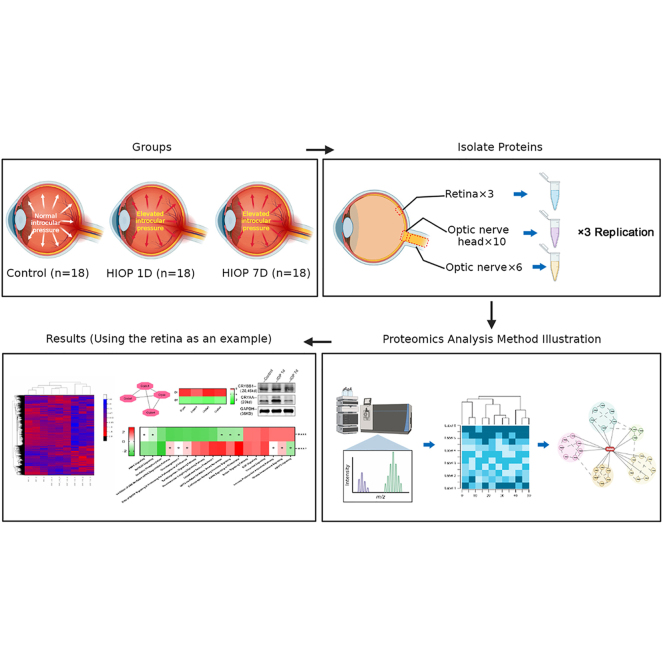

## In Brief

This study aimed to identify the early proteomic alterations in the retina, optic nerve head (ONH), and optic nerve (ON) after establishing a rat model of ocular hypertension (OHT). The protein expression patterns suggested that OHT caused *mitochondrial dysfunction in the retina, ONH, and ON tissues.* The inflammatory response is mainly changed in retina, and ECM/integrin/liposome complex is mainly changed in ONH. The study provided foundational insights into new target proteins and possible pathway mechanisms that need to be further investigated.

Glaucoma, which is a group of optic neuropathies characterized by progressive degeneration of retinal ganglion cells (RGCs), mainly results in optic nerve (ON) damage and visual field loss (1). Given that its cause is still not well-established and markers for early diagnosis and therapeutic targeting are lacking, glaucoma is becoming the leading cause of irreversible blindness globally, and the number of affected patients is estimated to reach 111.8 million by 2040 (2). Intraocular pressure (IOP) is considered the most important risk factor for glaucoma onset and progression. Previous studies have reported that the increase in IOP and induction of RGC loss may involve several molecular pathways, including axonal transport failure, neurotrophic factor deprivation, activation of intrinsic and extrinsic apoptotic signals, mitochondrial dysfunction, excitotoxic damage, dysfunction of reactive glia and loss of synaptic connectivity, oxidative stress, and neuroinflammation (3, 4, 5). Mehdi Mirzaei (6) developed an experimental glaucoma rat model and analyzed the retinal tissues’ protein expression changes using a multiplexed quantitative proteomics approach and highlighted the differential modulation of nuclear receptor signaling, cellular survival, protein synthesis, transport, and cellular assembly pathways. Liu *et al*. (7) found that the retinas of glaucomatous mice with normal IOP expressed amyloid deposition, RNA splicing, microglia activation, and reduction of crystallin production, similar to Alzheimer's disease.

However, the above-mentioned findings are mainly associated with the pathological stage when the RGCs are already damaged, and it is yet to be established whether they are pathogenic factors or accompanying events of glaucoma. Therefore, the question remains unanswered as to what molecular biological changes occur during the early pathological progression from pathological IOP elevation to the appearance of RGC lesions.

Increasing evidence also confirms that the pathological changes induced by elevated IOP in glaucoma have anatomical characteristics. Axonal transport in optic nerve head (ONH) is impaired and reaches its maximum damage after 24 h of IOP elevation, which may be due to reduced ATP availability. However, it is not until 3 days after IOP elevation that axonal cytoskeletal damage appears in the ON (8, 9). Disruption of astrocytic gap junctional communication was detected localized to the ONH 3 days after IOP elevation with no detected alterations in retina until 7 days (10). The alterations in microglia were initially detected in the ONH and further progress along the retina overtime (11). However, the early molecular events caused by elevated IOP in different anatomical sites have not been systematically studied. This study used an animal model of acute elevated intraocular hypertension to analyze molecular biological changes in three tissues (retina, ONH, and ON) on days 1 and 7 of ocular hypertension (OHT) (with or without RGC loss) using proteomics.

## Experimental Procedures

### Animals

Adult Male Sprague Dawley rats weighing between 220 to 250g were purchased from Charles River Company. The rats were kept in temperature-controlled rooms with a 12/12-h light/dark cycle and provided with standard food and water ad libitum. All experiments were performed following the ARVO Statement for the Use of Animals in Ophthalmic and Vision Research. The Institutional Animal Care and Use Committee of Capital Medical University, Beijing, approved this study (AEEI-2016-093 and AEEI-2021-252).

### Induction of OHT and Pressure Measurements

OHT was induced using a hydrogel injection model following a previous study (12). Rats were anesthetized with an intraperitoneal injection of 1% pentobarbital sodium (30 mg/kg). Hyaluronic acid-vinyl sulfone and hyaluronic acid-thiol group polymers were purchased from Pleryon Therapeutics. Five microliters of each sample were mixed on an ice-cold metal plate before injection. A paracentesis was performed at the limbal region to drain aqueous humor using a pulled glass micropipette needle. During this procedure, caution was exercised to avoid damaging the iris or lens. Subsequently, 1.5 μl of the mixture was aspirated using a pulled glass micropipette needle and injected into the anterior chambers of both eyes, with the anterior chamber angle as the target. And 1.5 μl of PBS (Kelun Pharmaceutical Co, Ltd) was injected into the anterior chambers of both eyes in the control group.

At baseline and during the following days, IOP was measured simultaneously (10:00–12:00 AM) on each day under anesthesia using a tonometer (TonoLab). The Tonolab-generated average was considered as one value, and we recorded three values for each eye. The mean of these three values was used as the IOP measurement.

### Quantification of RGCs

Retinal flat mounts were stained with antibodies to the RGC marker, RNA-binding protein with multiple splicing (RBPMS), and surviving RGCs were quantified (13). The extracted eyes were fixed in 4% paraformaldehyde for 30 min, and the retinas were separated and immersed in 0.5% Triton X-100 for 4h and 1% bovine serum albumin (A8010, Solarbio) for 2 h. After the retinas were incubated with RBPMS antibody (1:500, GTX118619, GeneTex) for 24 h and gently rinsed in PBS three times, they were incubated with secondary fluorescein antibody for 1 h in the dark at room temperature. The cell nuclei were counterstained with 4′,6-diamidino-2-phenylindole (ab104139, Abcam). Twelve nonoverlapping images from the entire retina were captured, and the ImageJ Multi-point Tool was used to count the RBPMS-positive RGCs. The counts of surviving RGCs in the experimental and control groups were compared to determine the survival of the RGCs (Control n = 3, IOP 1D n = 3, IOP 7D n = 3).

### Tissue Harvesting of the ONH, ON, and Retina for Proteomic Analysis

On day 1 and 7 after the injection, the rats were sacrificed under deep anesthesia, and their eyes were immediately enucleated. After the removal of the anterior segment, the sclera was clamped to turn the eyeball around, and the white retina was stripped from the choroid. After retinal dissection, a circular area of the retina with the optic disc as the center and a diameter of 1 to 1.5 mm was taken as the ONH. The initial (approximately 5 mm) portion of the ON was obtained.

### Protein Extraction

Protein extraction was carried out using a mammalian tissue cell protein extraction kit (Bangfei Biotechnology Co, Ltd), and the protein concentration was determined using a protein quantification kit (Dingguo Changsheng). Protein digestion was carried out using the filter-assisted sample preparation method (14). Briefly, 0.1 g of the tissue and 1 ml of the extraction buffer were subjected to probe sonication for 5 min, followed by centrifugation for 5 min in a 12,000-rpm centrifuge at room temperature, boiling for 5 min at 95 °C, and centrifugation at 20,000*g* at 25 °C. Protein quantification was performed using the Bradford method. Three biological replicates were done for each sample. Using the filter-assisted sample preparation approach, the protein samples were reduced and alkylated using DTT and iodoacetamide before being digested with trypsin. All of the peptide samples were collected for mass spectrometry analysis.

### Protein Enzymolysis and TMT Labeling

Peptides (70 μg) from each sample were labeled with TMT10plex Isobaric Label Reagent (Thermo Fisher Scientific) and analyzed using nano-liquid chromatography-tandem mass spectrometry with an ultrahigh-pressure LC system (EASY nLC 1000) and Q ExactiveTM Plus mass spectrometry. Peptide fragmentation was performed using the SPS-MS3 method described previously (15).

### Database Searching/Quantification

The proteins were quantified and identified using Proteome Discoverer (version 2.0). The spectra were searched in an indexed *Rattus norvegicus* UniProt database (January 2nd 2019; 36,097 sequences) using the Sequest search algorithm. The search results included the carbamidomethylation of cysteine residues and tandem mass tag (TMT) labeling of peptide N termini and lysine residues as static modifications, oxidation of methionine as a dynamic modification, and a precursor ion tolerance of 20 ppm and fragment ion tolerance of 0.8 Da. A linear discriminant analysis was used to filter the Sequest matches to a false discovery rate (FDR) of 0.01 at the peptide level based on matches to reversed sequences (16). The proteins were ranked by multiplying the peptide probabilities, and the dataset was finally filtered to 0.01 peptide FDR. The XCorr score was 1.2 and the database-filtering parameters are set according to the software default. The TMT reporter ion quantification was assessed using the strategy described previously (17, 18). Peptides with a total signal-to-noise ratio greater than 200 for all channels and a precursor isolation specificity greater than 0.75 were used for quantification. The TMT reporter ion intensity values were normalized by summing values across all peptides within each channel and correcting each channel to the same summed value. Protein level quantification was performed using normalized S/N values for all peptides assigned to a given protein. The reproducibility of the TMT experiment was further evaluated using “TMTPrepPro” software (ftp://ftp.proteome.org.au/TMTPrepPro) reported previously (19).

### Bioinformatics and Functional Pathway Analysis

STRING protein–protein network analysis was used for functional pathway enrichment and protein–protein interaction analysis of differential regulatory proteins. The STRING Cytoscape plugin was used to detect the protein–protein interactions, as well as classify the differentially modulated pathways and networks. We selected the top three significant modules based on the degree of importance. Ingenious pathway analysis (IPA) with the canonical pathway analysis was used to analyze the function-specific genes in each network. Absolute values of the z-score ≥1.5 and *p*-values <0.05 denoted significant differences, while values of −1.5 to 1.5 represented no significant change in the levels of expression.

### Western Blot

Retinas were collected using a radioimmunoprecipitation assay lysis buffer. The proteins were denatured by heating in a water bath at 100 °C for 5 min, separated by 15% SDS-PAGE electrophoresis, and transferred to polyvinylidene fluoride membranes (Millipore). The membranes were blocked with a 5% skimmed milk buffer for 1 h at room temperature and incubated overnight at 4 °C with specific antibodies against CRYAA (Abcam), CRYBB1 (Abcam), or GAPDH (Millipore). After washing, the membranes were incubated with the secondary antibody for 1h at room temperature. After rinsing, the proteins were detected by a ChemiDoc MP imaging system (Bio-Rad). The immunoblots were quantified by densitometry by ImageJ software (https://imagej.nih.gov/ij/index.html, v1.53, National Institutes of Health) and normalized to the GAPDH level.

### Experimental Design and Statistical Rationale

Forty-five rats were used for proteomics (control n = 15, IOP 1D n = 15, IOP 7D n = 15); both eyes were included. Due to the varying size of the tissue samples, each examination required 10 ONH samples, six ON samples, and three retina samples. The proteomics was conducted with three replicates, and each group consisted of five animals per replicate. All samples from baseline, 1 day and 7 days after HIOP were processed in the same laboratory environment.

For Western blot, nine rats were used (control n = 3, IOP 1D n = 3, IOP 7D n = 3), and both eyes were included too. Two retinas were mixed as one biological sample, and a total of three replicates were performed.

Statistical comparisons were performed using SPSS software (https://www.ibm.com/cn-zh/spss?lnk=flatitem, version 23.0, SPSS), and the GraphPad Prism (v8.0) was used to plot the diagrams. Results are expressed as mean ± SD. Differentially expressed proteins (DEPs) were assessed using a two-sample *t* test (*p* ≤ 0.05) and a fold change threshold (≥1.2 for upregulation or ≤0.83 for downregulation). Proteins with values of 0.833<FC<1.2 or *p* >0.05 were referred to as non-DEPs. IOP analysis was performed with independent samples *t* test or Mann–Whitney test dependent on whether the data conforms to the Gaussian distribution. The one-way ANOVAs were used for comparison of RGC number and the protein expression levels evaluated by Western blot among three groups. *p* < 0.05 was considered statistically significant.

## Results

### The OHT Model was Prepared by Anterior Chamber Infusion with Hydrogel

**Fig. 1:**
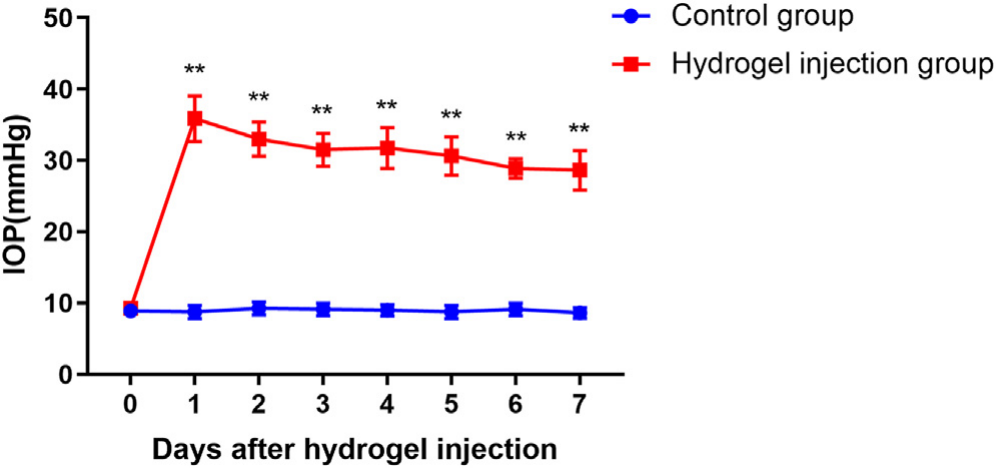
**Elevation of intraocular pressure after hydrogel infusion.** The mean IOP increased significantly from 9.3 ± 0.7 mmHg at baseline to 35.9 ± 3.2 mmHg on day 1 and 28.6 ± 2.8 mmHg on day 7. The IOPs of the controls did not change (mean ± SEM, n = 8, *p* < 0.001). ∗, *versus* control; ∗∗*p* ＜ 0.001. IOP, intraocular pressure.

The hydrogel infusion led to a significant increase in IOP in all animals. The IOP increased from a mean of 9.3 ± 0.7 mmHg to 35.9 ± 3.2 mmHg on day 1 after anterior aqueous humor gel infusion. On the following day, the IOP was stable. On day 7 of OHT, the IOP was 28.6 ± 2.8 mmHg and the IOP in control group remained at baseline levels (8.9 ± 0.8 mmHg, *p* < 0.001; Fig. 1).

**Fig. 2:**
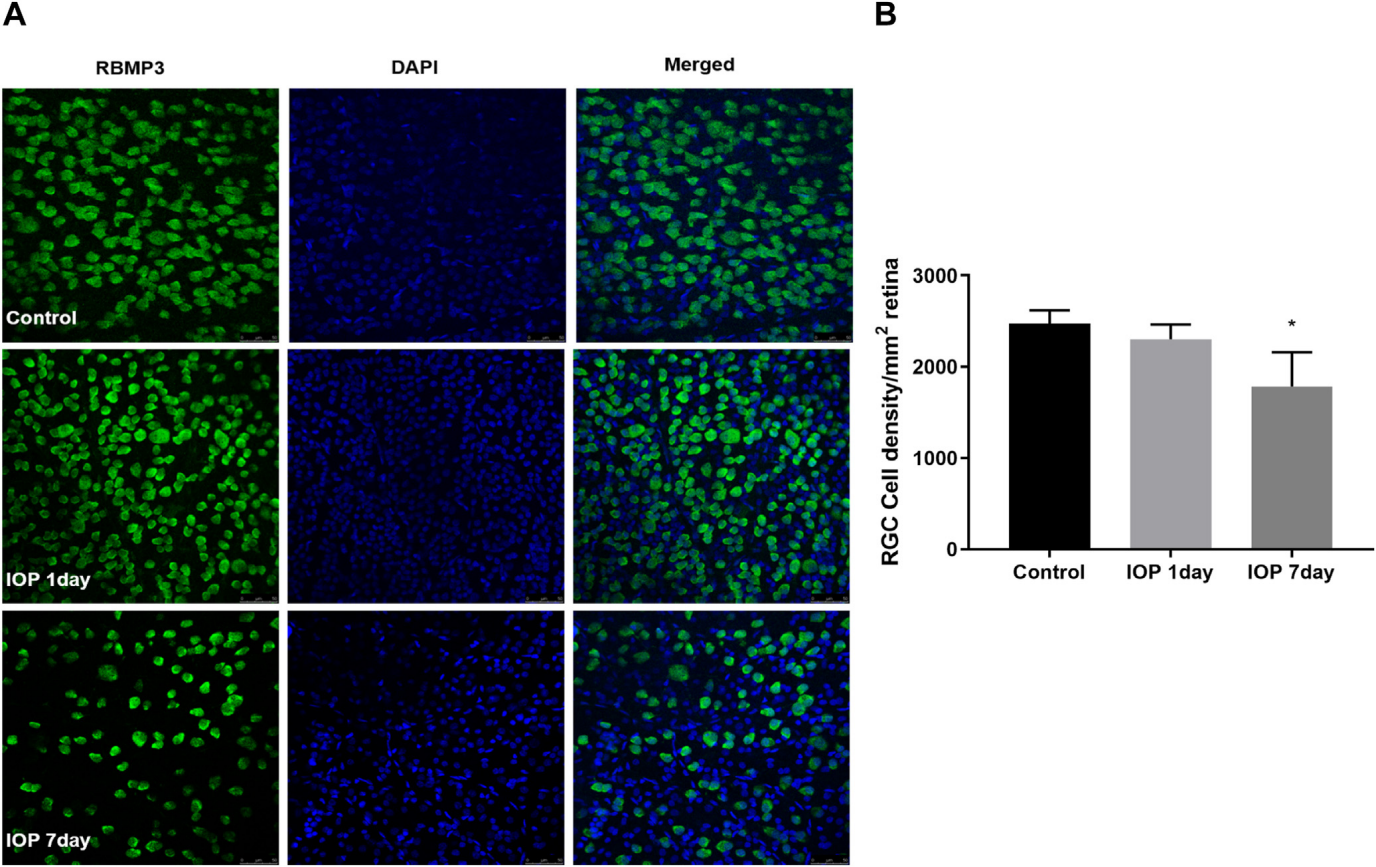
**Effect of elevated intraocular pressure on retinal morphology.***A*, double-labeling immunohistochemistry of DAPI and RBMP3 in the control on days 1 and 7 of elevated IOP (Scale bar represents 50 mm). *B*, retinal ganglion cells decreased on day 7 after elevated IOP (2474 ±84 RGCs/mm^2^*versus* 1782 ± 216 RGCs/mm^2^, *p* < 0.05). ∗, *versus* control; ∗*p* ＜ 0.05. IOP, intraocular pressure; RGC, retinal ganglion cell. DAPI, 4′,6-diamidino-2-phenylindole.

The elevation of IOP resulted in a progressive RGC loss over time. In the retinal tissue exposed to elevated IOP, the number of RGCs decreased after 1 day (2302 ± 93 RGC/mm2) of IOP elevation with no statistical significance. On day 7 of OHT, the number of RGCs decreased by 27.9% compared with that of the contralateral eye (2474 ± 84 RGC/mm2 *versus* 1782 ± 216 RGC/mm2, *p* < 0.05; Fig. 2), which is similar to the finding of previous study (12).

### Proteomics Overview

**Fig. 3:**
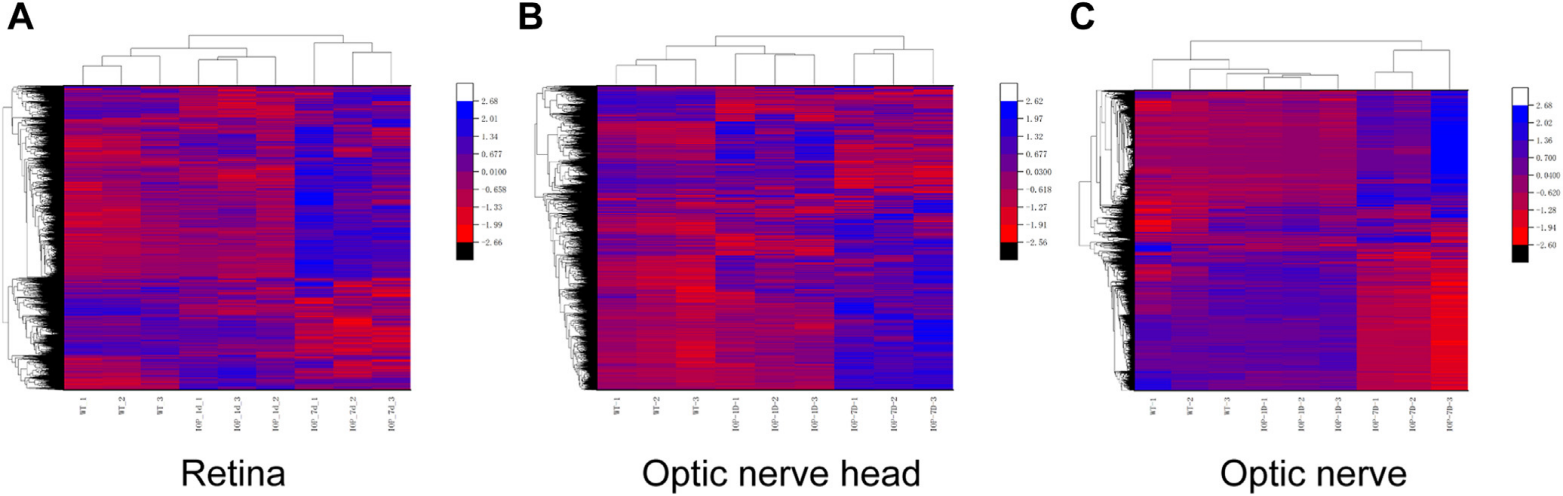
**Heatmap (hierarchical clustering) displaying protein variations in various ocular tissues.** This heatmap includes the retina (*A*), optic nerve head (*B*), and optic nerve (*C*) at different time points after ocular hypertension (day 1 and day 7). Each row represents one protein, and each column represents one sample. *Red* and *blue* indicate relative increases or decreases in protein abundance, respectively (WT: control sample; IOP_1d: day 1 after ocular hypertension; IOP_7d: day 7 after ocular hypertension).

The quality of the data and reproducibility of the biological replicates across groups were assessed using hierarchical clustering and statistical metrics. The protein distributions of the individual biological replicates of the control and glaucomatous eyes were significantly similar conditions, which confirmed the reproducibility of the data for further analysis (Fig. 3). A total of 6403 reproducible proteins were identified in the retinal tissue on day 1 of OHT (1% FDR), with 560 being differential proteins (representing 8.7% of the reproducible proteins). On day 7 of OHT, 4399 reproducible proteins were identified, with 489 being differential proteins (11.1% of the reproducible proteins). In the ONH tissue, a total of 5493 reproducible proteins were identified on day 1 of OHT (1% FDR), which included 428 differential proteins (7.8% of the reproducible proteins). On day 7 of OHT, 4544 reproducible proteins were identified, with 761 being differential proteins (16% of the reproducible proteins). In the ON tissue, 5455 reproducible proteins were determined on day 1 of OHT (1% FDR), including 257 differential proteins (4.7% of the reproducible proteins). On day 7 of OHT, 3835 reproducible proteins were identified, with 205 being differential proteins (5.3% of the reproducible proteins) (Table 1).

**Table 1:** The order of protein expression alterations (retina, optic nerve head, and optic nerve) according to the ratio of protein expression alteration

Ocular tissue	≥1.2 or ≤-1.2 (fold change)	Ratio (%)	Rankings
1 day after elevated IOP			
Retina	560/6403	8.7%	1
Optic nerve head	428/5493	7.8%	2
Optic nerve	257/5455	4.7%	3
7 days after elevated IOP			
Retina	489/4399	11.1%	2
Optic nerve head	761/4544	16%	1
Optic nerve	205/3835	5.3%	3

IOP, intraocular pressure.

### Expression of Universal Proteins in Different Tissues of the Elevated IOP Model

The DEPs that were universal in the three tissues on day 1 of OHT were associated with the complement coagulation cascade (Kng1, Orm1, Hp, Ttr, Kng2, Itih4, Cox7a2, Htra1, A2m, Upp1, Rpl8), ribosome biogenesis, and histone H2A/H2B/H3/H4 (Hp1bp3, Hist1h1d, H1f0, Hist1h1b, Rpl7a, Rpl26, Rpl14, Hist1h1c, Rpl24, Rpl21, Rpl36a, Rpl28, Rpl36, Rpl8, Rps4x, and Rpl27) and mitochondrial (Ndufa4, Hsbp1, Atp5o, Gstz1, Cdipt, PPIase, Atp5mj) dysfunction; the DEPs that were universal in the three tissues on day 7 of OHT were associated with the coagulation cascade (Igtp, Fth1, Clic1, A2m, Gss, Trmt112, Vwf) and the axonal synapses (Snap25, Rhoa, Mpc1).

### Differentially Regulated Protein Networks Between Day 1 and 7 of OHT in the Retina

**Fig. 4:**
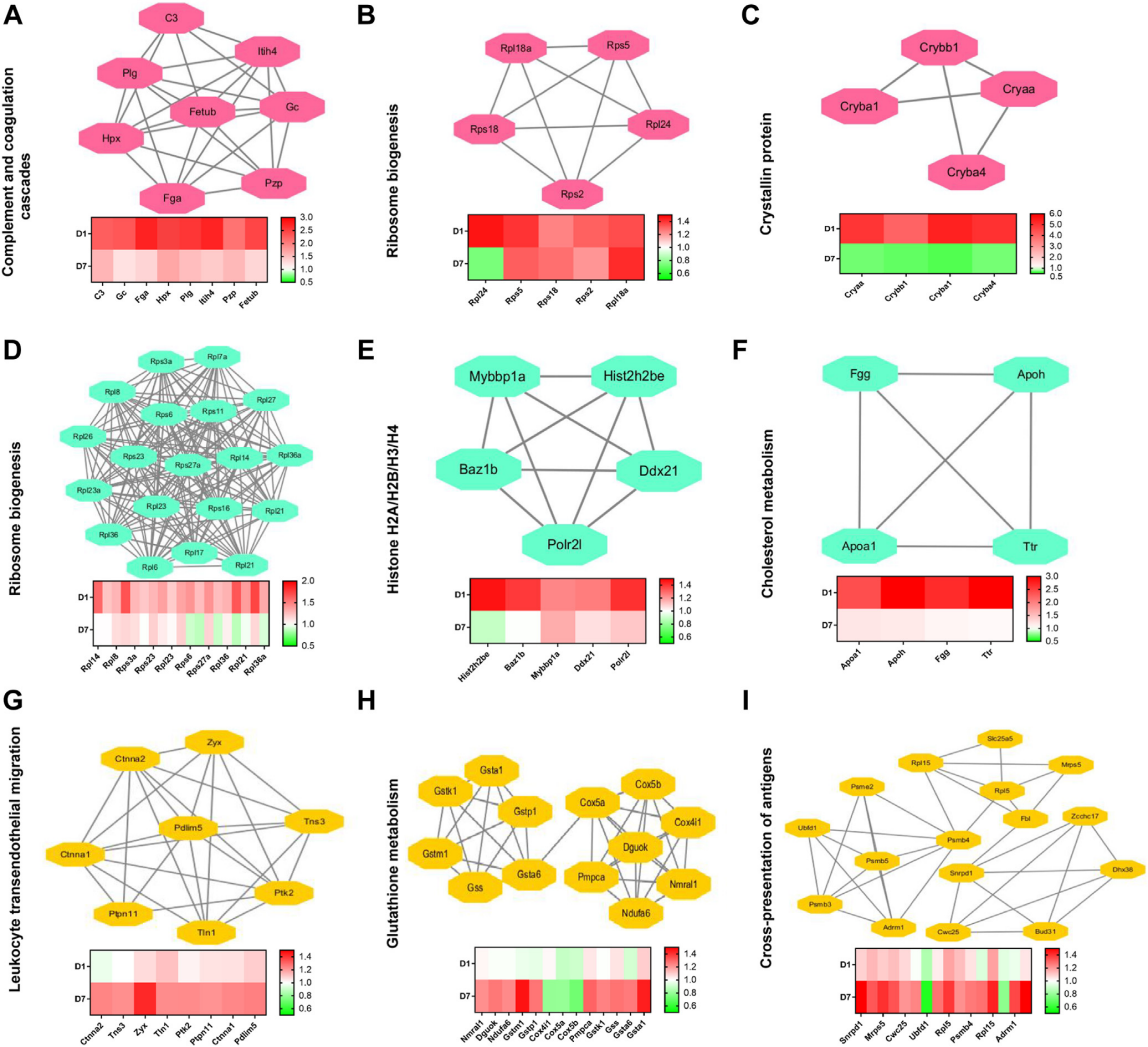
**Differentially regulated proteins between day1 and 7 in the glaucomatous retina.** Functional interaction networks analyzed by the String Cytoscape plugin indicate DEPs in expression changes on day 1 and 7 of OHT (*A*–*C*), DEPs changes on day 1 but restored to baseline on day 7 of OHT (*D*–*F*), and DEPs only changed on day 7 of OHT (*G*–*I*). The network nodes are labeled with gene symbols, and the corresponding fold changes are indicated below (*red*: upregulated; *green*: downregulated). DEP, differentially expressed protein; OHT, ocular hypertension.

Sixty-eight DEPs showed changes in expression on day 1 and 7 of OHT. These DEPs were related, and they included those involved in complement and coagulation cascades, ribosome biogenesis, and crystallin protein (Fig. 4, *A*–*C*). Among them, the most significant change in expression level are crystallins. A further 197 DEPs showed changes in expression on day 1 of OHT, but the expressions were restored to baseline levels on day 7 of OHT. These DEPs were involved in pathways including ribosome biogenesis, histone H2A/H2B/H3/H4, and cholesterol metabolism (Fig. 4, *D*–*F*). Finally, 354 DEPs that only changed in expression after 7-days OHT were involved in leukocyte transendothelial migration, glutathione metabolism/respiratory electron transfer, and cross-presentation of antigens (Fig. 4, *G*–*I*).

### Differentially Regulated Canonical Pathways in the Retina Between Day 1 and 7 of OHT

AMPK and RHOGDI signaling were not inhibited until day 7. Oxidative phosphorylation was inhibited on day 1 and 7 after OHT. The following were inhibited on day 1 but restored on day 7 of OHT: inhibition of ARE-mediated mRNA degradation, gluconeogenesis I, MAPK signaling for promoting the pathogenesis of influenza, nNOS signaling in neurons, coronavirus pathogenesis pathway, glycolysis I, insulin receptor signaling, TCA cycle II (eukaryotic), coronavirus replication pathway, and regulation of actin-based motility by Rho. The following nine pathways were inhibited on day 1 but activated on day 7 of OHT: synaptogenesis signaling pathway, neurovascular coupling signaling pathway, glutathione redox reactions I, xenobiotic metabolism general signaling pathway, polyamine regulation in colon cancer, glutathione-mediated detoxification, RHOA signaling, Reelin signaling in neurons, and Fcγ receptor–mediated phagocytosis in macrophages and monocytes.

Inositol biosynthesis was the most notable pathway that was not activated until day 7, and it included 3-phosphoinositide degradation, super pathway of inositol phosphate compounds, D-myo-inositol-5-phosphate metabolism, D-myo-inositol (3, 4, 5, 6)-tetrakisphosphate biosynthesis, D-myo-inositol (1, 4, 5, 6)-tetrakisphosphate biosynthesis, and 3-phosphoinositide biosynthesis. The next most notable pathway was Rho family GTPase signaling, followed by the nuclear factor erythroid 2–related factor 2 (NRF2)-mediated oxidative stress response, insulin secretion signaling pathway, 4-1BB signaling in T lymphocytes, actin cytoskeleton signaling, remodeling of epithelial adherence junctions, ERK/MAPK signaling, and CXCR4 signaling. The pathways that were activated on day 1 and 7 of OHT included LXR/RXR activation, Sirtuin signaling pathway, EGF signaling, thrombin signaling, estrogen receptor signaling, and acute phase response signaling. The pathways activated on day 1 but restored to WT levels on day 7 of OHT were CNTF signaling, UVA-induced MAPK signaling, regulation of cellular mechanics by Calpain protease, Gαi signaling, production of nitric oxide and reactive oxygen species in macrophages, GP6 signaling pathway, and EIF2 signaling pathway (supplemental Fig. S1).

### Differentially Regulated Protein Networks Between Day 1 and 7 of OHT in the ONH

**Fig. 5:**
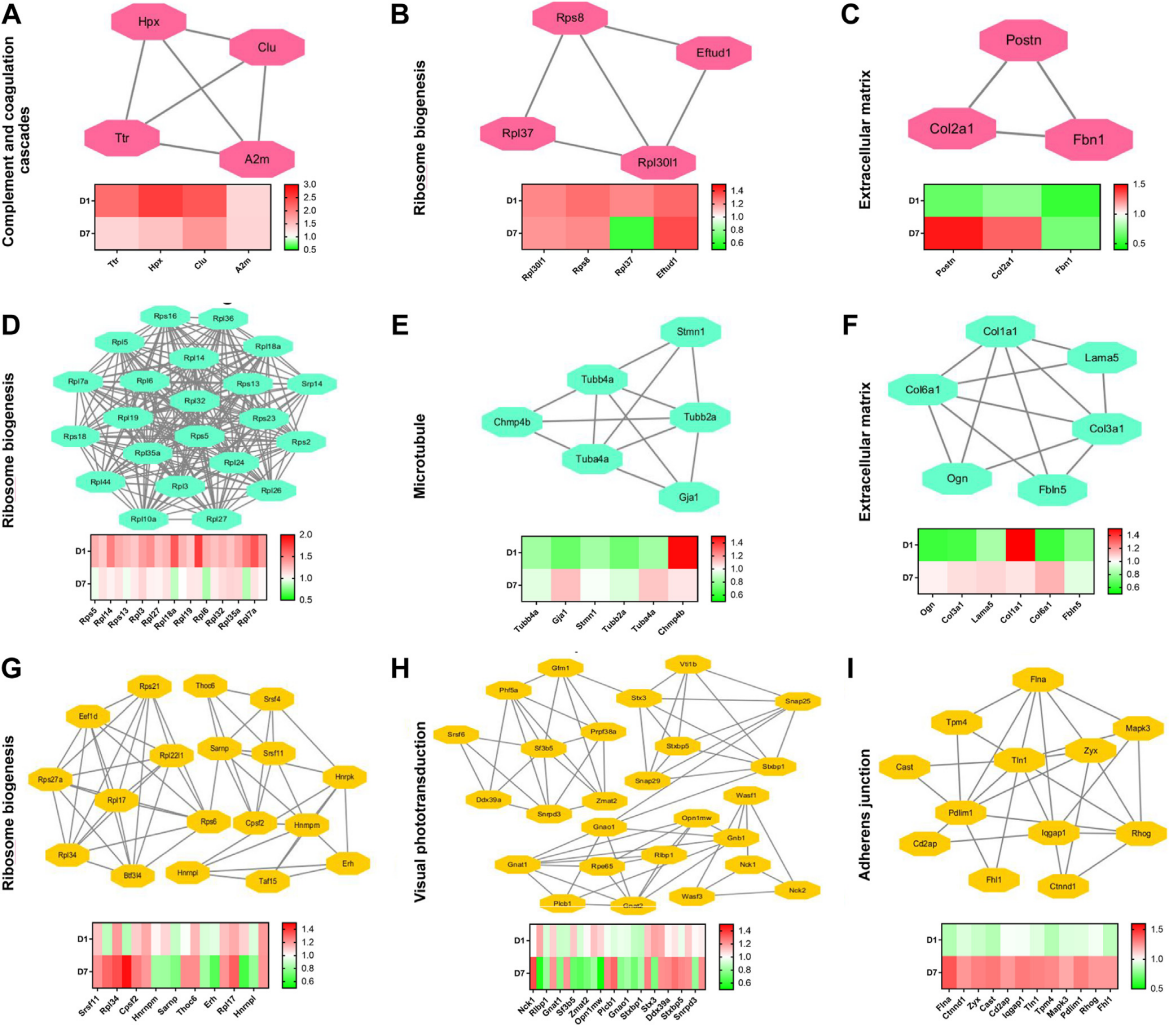
**Differentially regulated proteins between day1 and 7 in the glaucomatous optic nerve head.** Functional interaction networks analyzed by the String Cytoscape plugin indicate DEPs in expression changes on day 1 and 7 of OHT (*A*–*C*), DEP changes on day 1 but restored to baseline on day 7 of OHT (*D*–*F*), and DEPs only changed on day 7 of OHT (*G*–*I*). Network nodes are labeled with gene symbols, and the corresponding fold changes are indicated below. (*red*—upregulated; *green*—downregulated). DEP, differentially expressed protein; OHT, ocular hypertension.

Sixty-four DEPs showed changes in expression on day 1 and 7 of OHT, and they were found in separate interconnected networks including those involved in complement and coagulation cascades and ribosome biogenesis and in the extracellular matrix (Fig. 5, *A*–*C*). One hundred seventy-two DEPs showed changes in expression on day 1 but these were restored to WT level on day 7 of OHT; the networks identified included ribosome biogenesis, microtubules, and the extracellular matrix (Fig. 5, *D*–*F*). The networks of 525 DEPs that only showed changes in expression on day 7 were ribosome biogenesis, visual phototransduction, and the adherens junction (Fig. 5, *G*–*I*).

### Differentially Regulated Canonical Pathways in the ONH Between Day 1 and 7 of OHT

Among the pathways that were co-activated on day 1 and 7 of OHT, the most prominent was inositol biosynthesis, which involves 3-phosphoinositide degradation, D-myo-inositol-5-phosphate metabolism, super pathway of inositol phosphate compounds, D-myo-inositol (3, 4, 5, 6)-tetrakisphosphate biosynthesis, D-myo-inositol (1, 4, 5, 6)-tetrakisphosphate biosynthesis, 3-phosphoinositide biosynthesis, followed by insulin secretion signaling pathway, sphingosine-1-phosphate signaling, natural killer cell signaling, EIF2 signaling, UVA-induced MAPK signaling, synaptic long term depression, and endothelin-1 signaling. Six pathways were inhibited on day 1 and activated on day 7 of OHT, and they involved integrin signaling, tryptophan degradation X (mammalian, *via* tryptamine), GNRH signaling, paxillin signaling, epithelial adherens junction signaling, and leukocyte extravasation signaling. The pathways that were activated on day 1 but restored to WT level on day 7 of OHT were Sirtuin signaling pathway, NAD signaling pathway, estrogen receptor signaling, triacylglycerol biosynthesis, assembly of RNA polymerase III complex, and Semaphorin neuronal repulsive signaling pathway. The pathways that were inhibited on day 1 but restored to WT level on day 7 of OHT were coronavirus pathogenesis pathway, inhibition of ARE-mediated mRNA degradation pathway, PI3K/AKT signaling, glycolysis I, putrescine degradation III, GP6 signaling pathway, corticotropin releasing hormone signaling, and synaptic long term potentiation. Among the pathways that were not activated until day 7, including signaling by Rho family GTPases, Reelin signaling in neurons, synaptogenesis signaling pathway, cardiac hypertrophy signaling (Enhanced), Fcγ receptor–mediated phagocytosis in macrophages and monocytes, Ephrin receptor signaling, actin cytoskeleton signaling, remodeling of epithelial adherens junctions, thrombin signaling, and IL-8 signaling. The pathways that were not inhibited until day 7 included GPCR-mediated integration of enteroendocrine signaling exemplified by an L Cell, PTEN signaling, oxidative phosphorylation, PPAR signaling, PPARα/RXRα activation, and RHOGDI signaling (supplemental Fig. S2).

### Differentially Regulated Protein Networks Between Day 1 and 7 of OHT in the ON

**Fig. 6:**
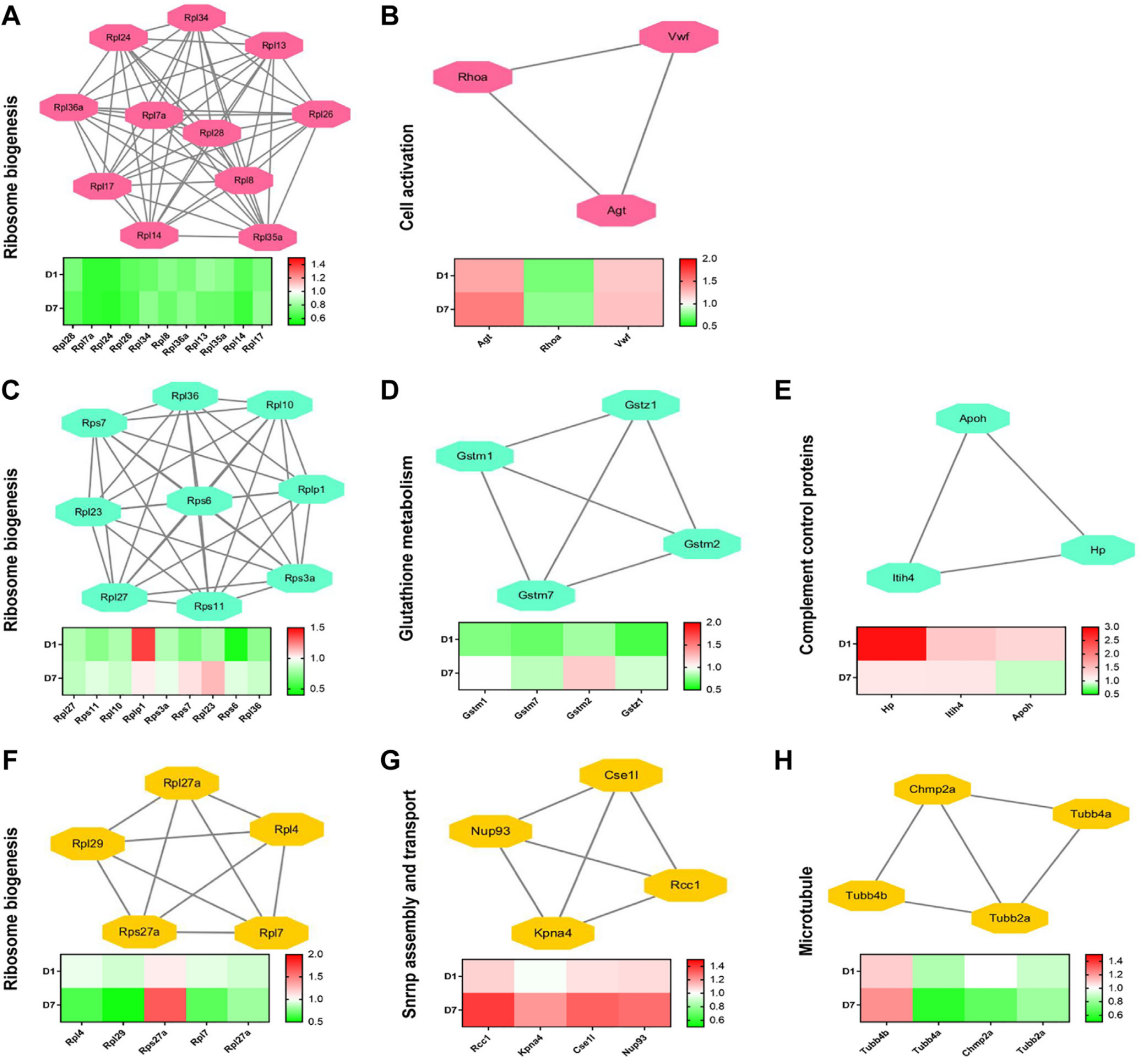
**Differentially regulated proteins between day1 and 7 in the glaucomatous optic nerve.** Functional interaction networks analyzed by the String Cytoscape plugin indicate DEPs in expression changes on day 1 and 7 of OHT (*A* and *B*), DEPs changes on day 1 but restored to baseline on day 7 of OHT (*C*–*E*), and DEPs only changed on day 7 of OHT (*F*–*H*). Network nodes are labeled with gene symbols, and the corresponding fold changes are indicated below. (*red*: upregulated; *green*: downregulated). DEP, differentially expressed protein; OHT, ocular hypertension.

Twenty-nine DEPs showed changes in expression on day 1 and 7 of OHT, and they were found in separate interconnected networks including those involved in ribosome biogenesis and cell activation (Fig. 6, *A* and *B*). Seventy DEPs showed changes in expression on day 1 but they were restored to WT level on day 7 of OHT; the networks identified included ribosome biogenesis, glutathione metabolism, and complement and coagulation cascades (Fig. 6, *C*–*E*). The networks of 140 DEPs that did not show changes in expression until day 7 were ribosome biogenesis, snRNP assembly and transport, and microtubules (Fig. 6, *F*–*H*).

### Differentially Regulated Canonical Pathways Between Day 1 and 7 of OHT in ON

Of the pathways that were co-activated on day 1 and 7 of OHT, the most prominent was inositol biosynthesis, which involves D-myo-inositol-5-phosphate metabolism, super pathway of inositol phosphate compounds, D-myo-inositol (3, 4, 5, 6)-tetrakisphosphate biosynthesis, D-myo-inositol (1, 4, 5, 6)-tetrakisphosphate biosynthesis, 3-phosphoinositide degradation, 3-phosphoinositide biosynthesis, followed by estrogen receptor signaling, regulation of the epithelial-mesenchymal transition by the pathway of growth factors, IL-6 signaling, acute phase response signaling, and IL-8 signaling. The pathways that were activated on day 1 but restored to WT level on day 7 after OHT were the synaptogenesis signaling pathway, NGF signaling, B cell activating factor signaling, and the activation of IRF by cytosolic pattern recognition receptors. The pathways inhibited on day 1 but restored to WT level on day 7 of OHT were the PTEN signaling, glutathione-mediated detoxification, super pathway of cholesterol biosynthesis, regulation of actin-based motility by Rho, cholesterol biosynthesis I, and antioxidant action of vitamin C. The pathways that were not activated until day 7 included the senescence pathway, UVB-induced MAPK signaling, CXCR4 signaling, IL-1 signaling, RANK signaling in osteoclasts, renin-angiotensin signaling, and pancreatic adenocarcinoma signaling. The only pathways that were co-inhibited on day 1 and day 7 of OHT were the nucleotide excision repair (supplemental Fig. S3).

### Validation of Crystallin Protein Expression in the Retina

**Fig. 7:**
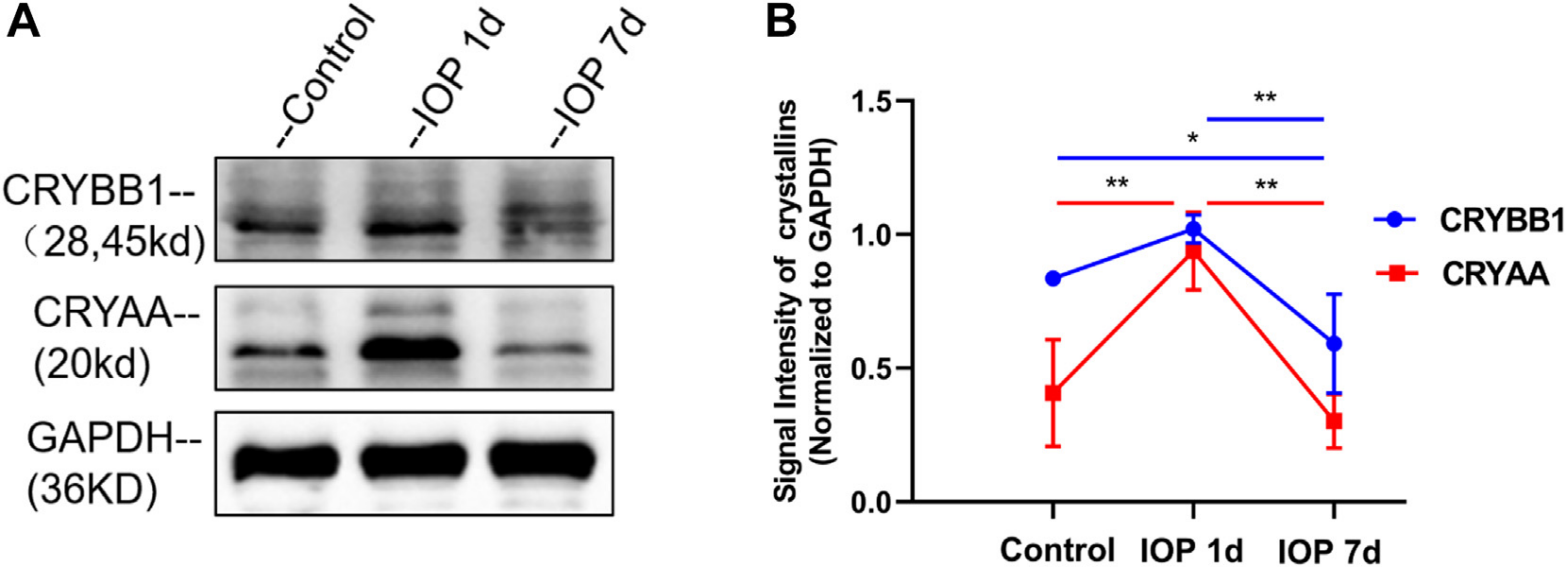
**Changes inalpha-crystallin A (CRYAA) and beta-crystallin B1 (CRYBB1).***A*, crystallin proteins including CRYAA and CRYBB1 were detected by Western blot. *B*, the relative protein expressions of CRYAA and CRYBB1 were normalized to GAPDH. The *red line* corresponds to CRYAA, while the *blue line* corresponds to CRYBB1; ∗*p* ＜ 0.05; ∗∗*p* ＜ 0.01.

Through String and Cytoscape analysis, we found that the FC value of crystallin proteins was the largest among all the protein networks, so we validated the changes in the expression of the crystallin protein. The expression of Alpha-crystallin A (CRYAA) and beta-crystallin B1 (CRYBB1) in the retina increased on day 1 after OHT, with CryAA showing a significant increase (CRYAA: 0.9375 ± 0.146 *versus* 0.4072 ± 0.200; *p* < 0.01. CRYBB1: 1.0205 ± 0.052 *versus* 0.8353 ± 0.022; *p* = 0.09). On day 7 after OHT, the expression of both proteins decreased significantly compared to day 1 (CRYAA: 0.3020 ± 0.102 *versus* 0.9375 ± 0.146, *p* < 0.01; CRYBB1: 0.5918 ± 0.185 *versus* 1.0205 ± 0.052, *p* < 0.01). Furthermore, the expression of CryBB1 on day 7 after OHT was significantly lower compared to the control group (0.5918 ± 0.185 *versus* 0.8353 ± 0.022; *p* < 0.05). Further details are provided in Figure 7.

## Discussion

Recently, multi-omics approaches have been used to investigate the etiology of glaucoma. However, most of these studies have focused on the intermediate and advanced stages of the disease characterized by RGC loss and have only evaluated the molecular changes at a single time point (20, 21). Glaucoma is a complex neurodegenerative disorder influenced by various factors, and a single time-point analysis cannot fully explain its underlying mechanisms or identify key regulatory targets or pathways. Thus, understanding the molecular changes that occur during the early pathological progression, from elevated IOP to RGC loss, is important for gaining insights into the regulatory mechanisms of glaucoma pathogenesis. In this study, we used proteomics to analyze the molecular changes at two critical time points during the early stages of elevated IOP: day 1 of OHT (without RGC loss) and day 7 of OHT (with RGC loss). The results showed that the molecular changes between these two time points were mainly concentrated in five pathways: mitochondrial dysfunction, endoplasmic reticulum (ER) stress, heat shock proteins, inflammation, and cytoskeletal and synaptic dysfunction.

### Heat Shock Proteins

Crystallins, which belong to the family of small heat shock proteins and comprise three major families (α, β, and γ crystallins), have been found within RGCs. They are thought to be produced by the activation of cellular defense mechanisms, indicating their involvement in injury and postinjury repair (22).

The String and Cytoscape interaction network analysis suggests that crystallines (CRYAA, CRYBA1, CRYBA4, and CRYBB1) are significantly higher at day 1 than on day 7. With the Western blot result, we found that protein expressions of CRYAA and CRYBB1 were significantly higher in the retina on day 1 and downregulated on day 7 of OHT.

CRYAA and CRYAB are the most studied members of the crystallin superfamily, and their molecular chaperone-like activity and ability to increase cellular resistance to oxidative stress-induced apoptosis are well established (23, 24). CRYBB1 is thought to be related with inflammatory diseases mediated by both cellular and humoral immunity (25). Previous studies have reported that the expressions of crystalline proteins alpha A, alpha B, betaA1, betaA4, betaB1, betaB2, betaB3, and gamma 4 genes were downregulated 2 weeks after IOP elevation, and the mRNA levels returned to control levels at 5 weeks after IOP elevation (22). The present study found that the expressions of these proteins were upregulated on day 1 and downregulated on day 7 of OHT, suggesting that crystalline proteins dynamics change during the early stages of glaucoma.

During the early stages of glaucoma, the activation of cellular defense mechanisms induces the upregulation of crystalline proteins expression and increases the ability to resist apoptosis in response to stress-induced cells (26). The early upregulation of crystallin reactivity is expected to protect RGCs from damage. However, it is gradually inhibited between 1 and 2 weeks after OHT and ultimately fails to protect RGCs from damage. The exact reasons for this decline are not yet fully understood. It is possible that the continuous stress and damage experienced by RGCs in glaucoma overwhelm the protective capabilities of crystalline proteins. Further research on the mechanisms of crystallin protection in glaucoma will help us to identify therapeutic targets that can bolster this protective response or introduce alternative protective mechanisms.

### Mitochondrial Dysfunction

Mitochondrial dysfunction has been closely associated with neurodegeneration in glaucoma patients and various glaucoma models, with RGCs being susceptible to mitochondrial function due to their high oxygen consumption (27). The mitochondria act as the primary generators of reactive oxygen species, which are primarily produced through electron leakage from complexes I and III of the oxidative phosphorylation pathway (28). The inhibition of electron transport through the oxidative phosphorylation pathway also increases the production of reactive oxygen species. Alterations in the proteome of the mitochondria of the ONH suggest impaired mitochondrial function, energy failure, and oxidative stress in glaucoma (29), which may indicate a state of vulnerability in which ganglion cells are prone to secondary insults they would otherwise be able to withstand (30).

We found that the expressions of mitochondria-related pathway proteins, namely Ndufa4, Atp5o, and Atp5mj, were downregulated in all three tissues after 1 day of OHT. The expressions of synaptic and mitochondria-related proteins such as Snap25 and Mpc1 were also downregulated after 7 days of OHT.

String and Cytoscape interaction network analysis suggested that the expression of glutathione metabolism/respiratory electron transport–related proteins (Gstm1, Gstp1, Gstk1, and Gsta1) was upregulated after 7 days of OHT mainly in the retina. It is well-documented that IOP increase in glaucoma induces oxidative stress in RGCs, and cells are protected from these effects of ROS by antioxidant enzymes, including superoxide dismutase, catalase, and glutathione transferase, and small molecule antioxidants such as glutathione. The expression of GSTM was downregulated by week 8 in a study by Mirzaei *et al* (31), while the expression of GSR, GSTA, GSTK, GSTM, GSTO, and GSTP were significantly upregulated in retinal tissues after 7 days of OHT in our study. A significant upregulation of carbonyl reductase 1 expression was observed. Glutathione sulfotransferase, one of the antioxidant enzymes, and carbonyl reductase 1, an NADPH-dependent oxidoreductase, were suggested to mediate neuroprotection against ischemic injury by reducing oxidative stress and neuroinflammation (32), indicating rescue effect on RGCs.

IPA analysis showed reduced oxidative phosphorylation in the retinal and optic disc regions after 1 day of OHT, with an increase in nrf2-mediated oxidative stress after 7 days of OHT. The transcription factor Nrf2 is a major regulator of redox homeostasis with potent antioxidant functions. Our study findings are consistent with those reported by Mirzaei *et al*. (6) on the proteomics of a high IOP 8-weeks model. Previous studies found that astrocytes can protect cocultured neurons from glutamate toxicity by expressing Nrf2 (33). These proteins are involved in regulating oxidoreductase activity and NAD binding, suggesting that a decline in mitochondrial energy supply after 1 day of OHT may trigger oxidative stress in the ONH and retina after 7 days of OHT. This implies that in glaucoma, a condition characterized by HIOP, there may be an early disruption in the energy supply to the mitochondria, which are the cellular powerhouses responsible for generating energy. This energy deficit could contribute to the development of oxidative stress, a state characterized by an imbalance between the production of harmful free radicals and the body's ability to neutralize them. Enhancing mitochondrial energy production may help mitigate the decline in energy supply observed in the early stages of OHT. Previous study found that decreased levels of NAD+ and mitochondrial dysfunction are the earliest molecular events observed in a mouse model of glaucoma. Furthermore, it has been shown that oral administration of vitamin B3 (NAD+ precursor nicotinamide) can prevent and protect RGC damage in the glaucoma mouse model (34).

### ER Stress

The ER is the primary organelle within cells that senses environmental changes, cellular stress, coordinates signaling pathways, and controls cellular function/survival (35). In experimental chronic glaucoma models, ER stress is involved in RGC death (36). To alleviate ER stress and restore ER homeostasis, cells activate a series of signaling pathways collectively known as the unfolded protein response (37). The three main molecular regulatory mechanisms underlying this response are pancreatic ER kinase (PERK), activating transcription factor 6, and inositol-requiring transmembrane kinase/endonuclease 1 (38). If these adaptive mechanisms are not sufficient to alleviate ER stress, the apoptotic program is initiated by the activation of CHOP or c-Jun N-terminal kinase (39).

During ER stress, unfolded proteins recruit BiP and separate it from PERK and activated PERK phosphorylates the alpha subunit of EIF2, rapidly and efficiently inhibiting translation initiation and reducing a load of newly synthesized proteins (40). String and Cytoscape interaction network analysis suggested that the EIF2 signaling pathway is associated with the activation of ribosome biogenesis–related proteins in the retina and ONH and is downregulated in the ON as early as on day 1 of OHT. The result suggests that ER stress may exist, and the mechanism or regulatory signal for the PERK, activating transcription factor 6, or inositol-requiring transmembrane kinase/endonuclease 1 needs to be further investigated.

The LXR/RXR activation signaling pathway was upregulated in retinal tissues but did not change significantly in ONH/ON tissues, and the estrogen receptor signaling pathway was upregulated in all three tissues on days 1 and 7 of OHT. Previous studies on glaucoma models have found that RXR activation can inhibit ER stress and protect RGCs (41), and estrogen receptors protect RGCs, astrocytes, and microglia by inhibiting ER stress (42). Therefore, there are numerous mechanisms involved in ER stress–induced apoptosis, and blocking only one cell death pathway involving the ER pathway may not be sufficient to keep cells alive. Identifying potential therapeutic targets beyond the ER stress–induced cell death pathway may also be important. Given the complexity of cellular responses to ER stress, further studies to explore other cellular pathways and processes involved in disease pathogenesis, such as inflammation or protein misfolding, may provide alternative avenues for therapeutic interventions.

### Inflammation

The present study found that the expression of alpha-2 macroglobulin (A2M) (ON: 1.29–1.34, ONH: 1.31–1.33, Retina: 1.39–1.29; *p* < 0.05) was upregulated in all three tissues on days 1 and 7 after OHT. Previously, A2M was considered a major component of the innate immune system; it acts as a broad-spectrum protease inhibitor, can be stimulated by the glucocorticoid receptor, stabilizes aberrant protein conformations to prevent aggregation, and serves as an acute-phase response protein in response to immune-mediated inflammatory reactions in central nervous system tissue (43). A previous report suggested that serum A2M concentrations are associated with preclinical Alzheimer's disease, reflecting early neuronal damage (44). In this study, we observed an increase in A2M expression after 1 day of OHT in three tissues. The changes were consistent across the tissues, with a fold change range of 1.29 to 1.39 (*p* < 0.05), indicating that A2M may serve as a potential biomarker for early neuronal damage in glaucoma. Further research focused on targeting A2M or the associated pathways has the potential to offer therapeutic benefits for glaucoma.

The String and Cytoscape interaction network analysis showed that the proteins associated with the complement and coagulation cascades were significantly upregulated on day 1 in all three tissues, most significantly in the retina (C3, Gc, Fga, Hpx, Plg, Itih4, Pzp, and Fetub). The increase in hemoglobin in the glaucomatous retina may reflect changes in the blood–brain (45) and blood–retinal barriers (46).

IPA analysis suggested that the acute phase response signals (STAT3) were upregulated in three tissues as early as after 1 day of OHT. A previous study found that phosphorylated STAT3 was markedly upregulated in astrocytes after a day of OHT before the reactivation of astrocytes, which can protect retinal neurons from damage (47). On day 7, the CXCR4 chemokine receptor signaling pathway was upregulated in all three tissues. Fcγ receptor signaling and leukocyte extravasation signaling were upregulated in RE and ONH tissues on day 7. The activation of the complement system occurs after 1 day of OHT, and fragments of the complement divide to attract and activate phagocytes in the retina, including microglia and macrophages. In conclusion, we found that the expressions of acute response proteins, such as complement proteins, macroglobulin, and glial cell activation signaling pathways, were mainly upregulated in the retina after 1 day of OHT, and the activation of T-cell homing signaling pathways and epithelial leukocyte transport pathways occurred after 7 days of OHT. The results provided insights into the immune response processes in different tissues during early-stage glaucoma. Previous studies (48) have already identified the protective role of inhibiting the immune response in early OHT, among which complement C3-targeted therapy has been studied the most (49). This study offers additional molecular targets and potential directions for further investigation.

### Cytoskeleton

A previous study reported that axonal dysfunction and degeneration in RGCs precede cell body loss (50). String and Cytoscape interaction network analysis identified microtubule-associated proteins in the ONH tissue (Tubb4a, Tubb2a, Tuba4a) that was downregulated on day 1 and recovered on day 7 of OHT. IPA analysis revealed that ONH and ON tissue inositol signaling pathways (including INPP5B, INPP4A, INPP4B, and PTEN) appeared to be upregulated on the first day, while RE tissues showed no significant differences. By day 7, all three tissues showed significant upregulation, especially in the ONH and retinal tissues. We also found that the signaling of Rho family GTPases and G-protein coupled receptor signaling were minimally inhibited after 1 day of OHT in the ONH and retinal tissue but upregulated on day 7 of OHT. The synaptogenic signaling pathways, integrins, and actin cytoskeletal signaling pathways were significantly upregulated in both retinal and ONH tissues after 7 days of OHT.

PtdIns (4, 5)P2 was regulated by kinases, including INPP5B, INPP4A, INPP4B, and PTEN, which acts as a major actin cytoskeleton and endocytic regulator. Actin dynamics are regulated by different small G-proteins (Rho, Rac, Cdc42, Arf) whose activities are modulated by PtdIns (4, 5)P2 (51). Previous studies have suggested that bidirectional communication between cells and the extracellular environment is usually mediated by several classes of cell surface receptors, of which integrins are the most abundant receptor family, and cells can sense and respond to changes in the microenvironment and convert mechanical signals into biological signals through integrin-dependent adhesion patch-mediated connections between the extracellular matrix and the actin skeleton (52, 53, 54).

The ECM/integrin/liposome complex can regulate several biological processes, including extracellular matrix deposition, remodeling processes, alterations in actin fiber contraction and alignment, and disturbances in the regulation of cell adhesion, and plays a role in the pathophysiology of glaucoma, particularly at the ONH (55). Our observation is consistent with the reports of previous studies that the ONH is the main site of axonal transport failure and cytoskeleton accumulation, and early changes are dominantly associated with tubulin, while late migration is dominantly associated with actin (56). We hypothesize that an accelerated turnover of the phosphoinositides may play a key role in mediating cellular responses to external stimuli.

## Conclusion

The protein expression patterns suggested that OHT caused *mitochondrial dysfunction in the retina, ONH, and ON tissues. ER stress and acute* phase *response* activation were observed after 1 day of OHT, and the protective mechanisms were upregulated after day 7 of OHT according to the protein expression patterns which including antioxidant enzymes and NRF2 pathway. The A2m protein was upregulated across two time points and three tissues stably, which may be a marker of early glaucoma damage. The mainly change between the two time points after elevated IOP in ONH are ECM/integrin pathways. We also validated this proteomic result of crystallin proteins with Western blot and found that protein expressions of CRYAA and CRYBB1 were significantly higher in the retina on day 1 and downregulated on day 7 of OHT, which are promising as therapeutic targets. The findings of the study provide foundational insights into new target proteins and possible pathway mechanisms that should be further investigated in future studies on the pathological mechanisms of glaucoma.

## Data Availability

The mass spectrometry proteomics data have been deposited to the ProteomeXchange Consortium (http://proteomecentral.proteomexchange.org) *via* the iProX partner repository (57, 58) with the dataset identifier PXD039972.

## Supplemental data

This article contains supplemental data.

## Conflict of interest

The authors declare no competing interests.

## Supplementary Data

Supplementary Fig. S3

CWS_Editorial_Certificate
