# Supplementary material for: Early Proteomic Characteristics and Changes in the Optic Nerve Head, Optic Nerve, and Retina in a Rat Model of Ocular Hypertension
Source: Mol Cell Proteomics. 2023 Oct 2;22(11):100654. doi: 10.1016/j.mcpro.2023.100654 (PMC10665672; doi:10.1016/j.mcpro.2023.100654)
Supplement: Supplementary Fig. S1 [file mmc4.pdf]

Figure S1

		DAY 1	DAY 7
1N7I	AMPK Signaling	*	
	RHOGDI Signaling	*	
1I7I	Oxidative Phosphorylation		
1I7N	Inhibition of ARE-Mediated mRNA Degrad		*
	Gluconeogenesis I		*
	Role of MAPK Signaling in Promoting the		*
	nNOS Signaling in Neurons		*
	Coronavirus Pathogenesis Pathway		*
	Glycolysis I		*
	Insulin Receptor Signaling		*
	TCA Cycle II (Eukaryotic)		*
	Coronavirus Replication Pathway		*
	Regulation of Actin-based Motility by Rho		*
1Y7A	Synaptogenesis Signaling Pathway		
	Neurovascular Coupling Signaling Pathwa		
	Glutathione Redox Reactions I		
	Xenobiotic Metabolism General Signaling		
	Polyamine Regulation in Colon Cancer		
	Glutathione-mediated Detoxification		
	RHOA Signaling		
	Reelin Signaling in Neurons		
	Fcγ Receptor-mediated Phagocytosis in M		
1N7A	NRF2-mediated Oxidative Stress Respons	*	
	Corticotropin Releasing Hormone Signalin	*	
	RANK Signaling in Osteoclasts	*	
	Nitric Oxide Signaling in the Cardiovascula	*	
	D-myo-inositol (3,4,5,6)-tetrakisphosphate	*	
	D-myo-inositol (1,4,5,6)-Tetrakisphosphate	*	
	Role of NFAT in Cardiac Hypertrophy	*	
	HIF1α Signaling	*	
	CCR3 Signaling in Eosinophils	*	
	LPS-stimulated MAPK Signaling	*	
	Purine Nucleotides De Novo Biosynthesis	*	
	Colanic Acid Building Blocks Biosynthesis	*	
	3-phosphoinositide Biosynthesis	*	
	PAK Signaling	*	
	Epithelial Adherens Junction Signaling	*	
	BMP signaling pathway	*	
	Remodeling of Epithelial Adherens Junctio	*	
	Pyridoxal 5'-phosphate Salvage Pathway	*	
	Xenobiotic Metabolism CAR Signaling Pat	*	
	RAC Signaling	*	
	Cardiac Hypertrophy Signaling	*	
	Chemokine Signaling	*	
	ERBB Signaling	*	
	14-3-3-mediated Signaling	*	
	Salvage Pathways of Pyrimidine Ribonucle	*	
	Adrenomedullin signaling pathway	*	
	Dopamine-DARPP32 Feedback in cAMP S	*	
	Xenobiotic Metabolism PXR Signaling Pat	*	
	Apelin Adipocyte Signaling Pathway	*	
	IL-1 Signaling	*	
	Renal Cell Carcinoma Signaling	*	
	Signaling by Rho Family GTPases	*	
	P2Y Purigenic Receptor Signaling Pathwa	*	
	D-myo-inositol-5-phosphate Metabolism	*	
	Synaptic Long Term Potentiation	*	
	eNOS Signaling	*	
	fMLP Signaling in Neutrophils	*	
	Integrin Signaling	*	
	GDNF Family Ligand-Receptor Interaction	*	
	Neurotrophin/TRK Signaling	*	
	D-myo-inositol (1,3,4)-trisphosphate Biosy	*	
	Pentose Phosphate Pathway	*	
	Leptin Signaling in Obesity	*	
	4-1BB Signaling in T Lymphocytes	*	
	D-myo-inositol (1,4,5)-trisphosphate Degra	*	
	Renin-Angiotensin Signaling	*	
	HGF Signaling	*	
	Insulin Secretion Signaling Pathway	*	
	PFKFB4 Signaling Pathway	*	
	Superpathway of D-myo-inositol (1,4,5)-tri	*	
	Ephrin Receptor Signaling	*	
	BER (Base Excision Repair) Pathway	*	
	GNRH Signaling	*	
	tRNA Charging	*	
	Superpathway of Inositol Phosphate Comp	*	
	Xenobiotic Metabolism AHR Signaling Pat	*	
	3-phosphoinositide Degradation	*	
	Role of PKR in Interferon Induction and An	*	
	Cardiac Hypertrophy Signaling (Enhanced	*	
	Aldosterone Signaling in Epithelial Cells	*	
	FGF Signaling	*	
	Tryptophan Degradation III (Eukaryotic)	*	
	Glutaryl-CoA Degradation	*	
	GPCR-Mediated Nutrient Sensing in Enter	*	
	Oxytocin Signaling Pathway	*	
	Paxillin Signaling	*	
	ERK/MAPK Signaling	*	
	Melatonin Signaling	*	
	CXCR4 Signaling	*	
	Spliceosomal Cycle	*	
	Gαs Signaling	*	
	IL-8 Signaling	*	
	Actin Cytoskeleton Signaling	*	
	Gαq Signaling	*	
	CD40 Signaling	*	
	Cholecystokinin/Gastrin-mediated Signalin	*	
	FLT3 Signaling in Hematopoietic Progenito	*	
	Pyrimidine Ribonucleotides De Novo Biosy	*	
	HER-2 Signaling in Breast Cancer	*	
	Endocannabinoid Neuronal Synapse Path	*	
	IL-6 Signaling	*	
1A7A	Sirtuin Signaling Pathway		
	EGF Signaling		
	Thrombin Signaling		
	Estrogen Receptor Signaling		
	Acute Phase Response Signaling		
	LXR/RXR Activation		
1A7N	Intrinsic Prothrombin Activation Pathway		*
	Glioma Invasiveness Signaling		*
	HIPPO signaling		*
	Regulation of eIF4 and p70S6K Signaling		*
	p70S6K Signaling		*
	Huntington's Disease Signaling		*
	CNTF Signaling		*
	UVA-Induced MAPK Signaling		*
	Regulation of Cellular Mechanics by Calpa		*
	Gαi Signaling		*
	Production of Nitric Oxide and Reactive O		*
	GP6 Signaling Pathway		*
	EIF2 Signaling		*

Figure S1: The canonical pathways enriched from IPA analysis of differentially regulated proteins in retina (glaucoma vs control). Red and green indicate relative increases or decreases in functional enrichment, respectively. The asterisks (*) indicate z-score values of -1.5 to 1.5 and p-values < 0.05 which represented no significant. IPA, ingenuity pathway analysis.
