# Supplementary material for: Early Proteomic Characteristics and Changes in the Optic Nerve Head, Optic Nerve, and Retina in a Rat Model of Ocular Hypertension
Source: Mol Cell Proteomics. 2023 Oct 2;22(11):100654. doi: 10.1016/j.mcpro.2023.100654 (PMC10665672; doi:10.1016/j.mcpro.2023.100654)
Supplement: Supplementary Fig. S2 [file mmc5.pdf]

Figure S2

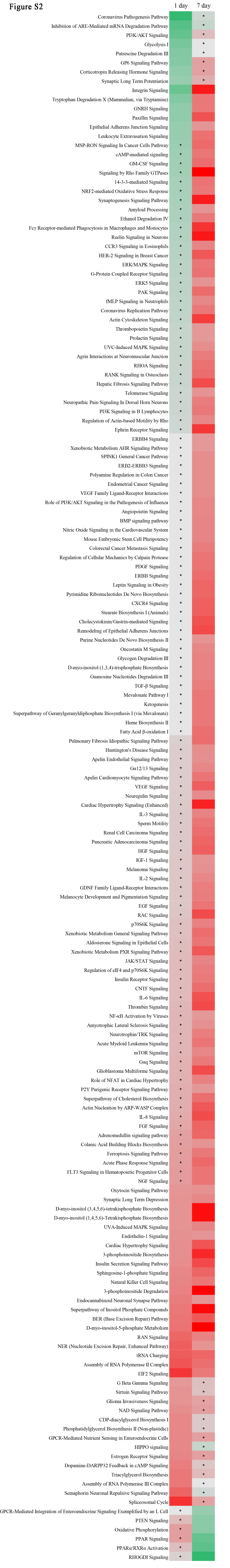
1 day
7 day
Coronavirus Pathogenesis Pathway
Inhibition of ARE-Mediated mRNA Degradation Pathway
PI3K/AKT Signaling
Glycolysis I
Putrescine Degradation III
GP6 Signaling Pathway
Corticotropin Releasing Hormone Signaling
Synaptic Long Term Potentiation
Integrin Signaling
Tryptophan Degradation X (Mammalian, via Tryptamine)
GNRH Signaling
Paxillin Signaling
Epithelial Adherens Junction Signaling
Leukocyte Extravasation Signaling
MSP-RON Signaling In Cancer Cells Pathway
cAMP-mediated signaling
GM-CSF Signaling
Signaling by Rho Family GTPases
14-3-3-mediated Signaling
NRF2-mediated Oxidative Stress Response
Synaptogenesis Signaling Pathway
Amyloid Processing
Ethanol Degradation IV
Fcγ Receptor-mediated Phagocytosis in Macrophages and Monocytes
Reelin Signaling in Neurons
CCR3 Signaling in Eosinophils
HER-2 Signaling in Breast Cancer
ERK/MAPK Signaling
G-Protein Coupled Receptor Signaling
ERK5 Signaling
PAK Signaling
fMLP Signaling in Neutrophils
Coronavirus Replication Pathway
Actin Cytoskeleton Signaling
Thrombopoietin Signaling
Prolactin Signaling
UVC-Induced MAPK Signaling
Agrin Interactions at Neuromuscular Junction
RHOA Signaling
RANK Signaling in Osteoclasts
Hepatic Fibrosis Signaling Pathway
Telomerase Signaling
Neuropathic Pain Signaling In Dorsal Horn Neurons
PI3K Signaling in B Lymphocytes
Regulation of Actin-based Motility by Rho
Ephrin Receptor Signaling
ERBB4 Signaling
Xenobiotic Metabolism AHR Signaling Pathway
SPINK1 General Cancer Pathway
ERB2-ERBB3 Signaling
Polyamine Regulation in Colon Cancer
Endometrial Cancer Signaling
VEGF Family Ligand-Receptor Interactions
Role of PI3K/AKT Signaling in the Pathogenesis of Influenza
Angiopoietin Signaling
BMP signaling pathway
Nitric Oxide Signaling in the Cardiovascular System
Mouse Embryonic Stem Cell Pluripotency
Colorectal Cancer Metastasis Signaling
Regulation of Cellular Mechanics by Calpain Protease
PDGF Signaling
ERBB Signaling
Leptin Signaling in Obesity
Pyrimidine Ribonucleotides De Novo Biosynthesis
CXCR4 Signaling
Stearate Biosynthesis I (Animals)
Cholecystokinin/Gastrin-mediated Signaling
Remodeling of Epithelial Adherens Junctions
Purine Nucleotides De Novo Biosynthesis II
Oncostatin M Signaling
Glycogen Degradation III
D-myo-inositol (1,3,4)-trisphosphate Biosynthesis
Guanosine Nucleotides Degradation III
TGF-β Signaling
Mevalonate Pathway I
Ketogenesis
Superpathway of Geranylgeranyldiphosphate Biosynthesis I (via Mevalonate)
Heme Biosynthesis II
Fatty Acid β-oxidation I
Pulmonary Fibrosis Idiopathic Signaling Pathway
Huntington's Disease Signaling
Apelin Endothelial Signaling Pathway
Gα12/13 Signaling
Apelin Cardiomyocyte Signaling Pathway
VEGF Signaling
Neuregulin Signaling
Cardiac Hypertrophy Signaling (Enhanced)
IL-3 Signaling
Sperm Motility
Renal Cell Carcinoma Signaling
Pancreatic Adenocarcinoma Signaling
HGF Signaling
IGF-1 Signaling
Melanoma Signaling
IL-2 Signaling
GDNF Family Ligand-Receptor Interactions
Melanocyte Development and Pigmentation Signaling
EGF Signaling
RAC Signaling
p70S6K Signaling
Xenobiotic Metabolism General Signaling Pathway
Aldosterone Signaling in Epithelial Cells
Xenobiotic Metabolism PXR Signaling Pathway
JAK/STAT Signaling
Regulation of eIF4 and p70S6K Signaling
Insulin Receptor Signaling
CNTF Signaling
IL-6 Signaling
Thrombin Signaling
NF-κB Activation by Viruses
Amyotrophic Lateral Sclerosis Signaling
Neurotrophin/TRK Signaling
Acute Myeloid Leukemia Signaling
mTOR Signaling
Gαq Signaling
Glioblastoma Multiforme Signaling
Role of NFAT in Cardiac Hypertrophy
P2Y Purigenic Receptor Signaling Pathway
Superpathway of Cholesterol Biosynthesis
Actin Nucleation by ARP-WASP Complex
IL-8 Signaling
FGF Signaling
Adrenomedullin signaling pathway
Colanic Acid Building Blocks Biosynthesis
Ferroptosis Signaling Pathway
Acute Phase Response Signaling
FLT3 Signaling in Hematopoietic Progenitor Cells
NGF Signaling
Oxytocin Signaling Pathway
Synaptic Long Term Depression
D-myo-inositol (3,4,5,6)-tetrakisphosphate Biosynthesis
D-myo-inositol (1,4,5,6)-Tetrakisphosphate Biosynthesis
UVA-Induced MAPK Signaling
Endothelin-1 Signaling
Cardiac Hypertrophy Signaling
3-phosphoinositide Biosynthesis
Insulin Secretion Signaling Pathway
Sphingosine-1-phosphate Signaling
Natural Killer Cell Signaling
3-phosphoinositide Degradation
Endocannabinoid Neuronal Synapse Pathway
Superpathway of Inositol Phosphate Compounds
BER (Base Excision Repair) Pathway
D-myo-inositol-5-phosphate Metabolism
RAN Signaling
NER (Nucleotide Excision Repair, Enhanced Pathway)
tRNA Charging
Assembly of RNA Polymerase II Complex
EIF2 Signaling
G Beta Gamma Signaling
Sirtuin Signaling Pathway
Glioma Invasiveness Signaling
NAD Signaling Pathway
CDP-diacylglycerol Biosynthesis I
Phosphatidylglycerol Biosynthesis II (Non-plastidic)
GPCR-Mediated Nutrient Sensing in Enteroendocrine Cells
HIPPO signaling
Estrogen Receptor Signaling
Dopamine-DARPP32 Feedback in cAMP Signaling
Triacylglycerol Biosynthesis
Assembly of RNA Polymerase III Complex
Semaphorin Neuronal Repulsive Signaling Pathway
Spliceosomal Cycle
GPCR-Mediated Integration of Enteroendocrine Signaling Exemplified by an L Cell
PTEN Signaling
Oxidative Phosphorylation
PPAR Signaling
PPARα/RXRα Activation
RHOGDI Signaling

Figure S2: The canonical pathways enriched from IPA analysis of differentially regulated proteins in optic nerve head (glaucoma vs control). Red and green indicate relative increases or decreases in functional enrichment, respectively. The asterisks (*) indicate z-score values of -1.5 to 1.5 and p-values < 0.05 which represented no significant. IPA, ingenuity pathway analysis.
