# Supplementary material for: Early Proteomic Characteristics and Changes in the Optic Nerve Head, Optic Nerve, and Retina in a Rat Model of Ocular Hypertension
Source: Mol Cell Proteomics. 2023 Oct 2;22(11):100654. doi: 10.1016/j.mcpro.2023.100654 (PMC10665672; doi:10.1016/j.mcpro.2023.100654)
Supplement: Supplementary Fig. S3 [file mmc6.pdf]

Figure S3

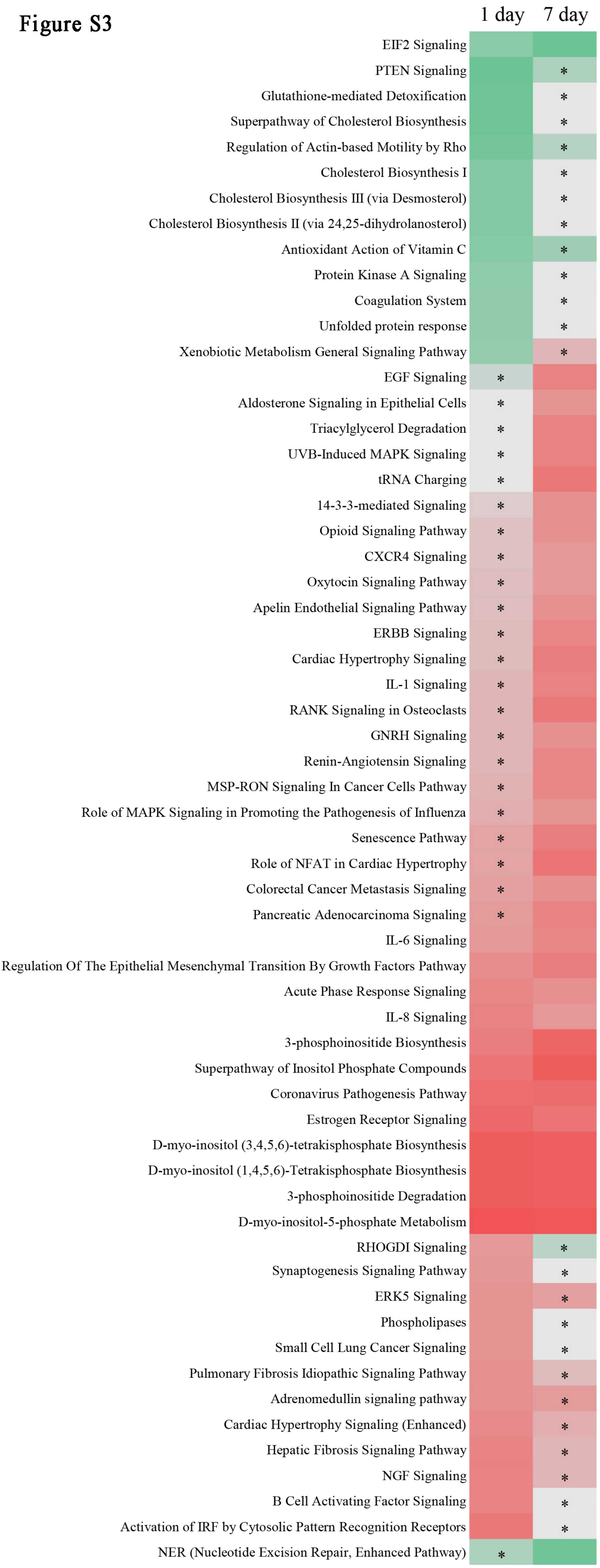

1 day
7 day
EIF2 Signaling
PTEN Signaling
Glutathione-mediated Detoxification
Superpathway of Cholesterol Biosynthesis
Regulation of Actin-based Motility by Rho
Cholesterol Biosynthesis I
Cholesterol Biosynthesis III (via Desmosterol)
Cholesterol Biosynthesis II (via 24,25-dihydrolanosterol)
Antioxidant Action of Vitamin C
Protein Kinase A Signaling
Coagulation System
Unfolded protein response
Xenobiotic Metabolism General Signaling Pathway
EGF Signaling
Aldosterone Signaling in Epithelial Cells
Triacylglycerol Degradation
UVB-Induced MAPK Signaling
tRNA Charging
14-3-3-mediated Signaling
Opioid Signaling Pathway
CXCR4 Signaling
Oxytocin Signaling Pathway
Apelin Endothelial Signaling Pathway
ERBB Signaling
Cardiac Hypertrophy Signaling
IL-1 Signaling
RANK Signaling in Osteoclasts
GNRH Signaling
Renin-Angiotensin Signaling
MSP-RON Signaling In Cancer Cells Pathway
Role of MAPK Signaling in Promoting the Pathogenesis of Influenza
Senescence Pathway
Role of NFAT in Cardiac Hypertrophy
Colorectal Cancer Metastasis Signaling
Pancreatic Adenocarcinoma Signaling
IL-6 Signaling
Regulation Of The Epithelial Mesenchymal Transition By Growth Factors Pathway
Acute Phase Response Signaling
IL-8 Signaling
3-phosphoinositide Biosynthesis
Superpathway of Inositol Phosphate Compounds
Coronavirus Pathogenesis Pathway
Estrogen Receptor Signaling
D-myo-inositol (3,4,5,6)-tetrakisphosphate Biosynthesis
D-myo-inositol (1,4,5,6)-Tetrakisphosphate Biosynthesis
3-phosphoinositide Degradation
D-myo-inositol-5-phosphate Metabolism
RHOGDI Signaling
Synaptogenesis Signaling Pathway
ERK5 Signaling
Phospholipases
Small Cell Lung Cancer Signaling
Pulmonary Fibrosis Idiopathic Signaling Pathway
Adrenomedullin signaling pathway
Cardiac Hypertrophy Signaling (Enhanced)
Hepatic Fibrosis Signaling Pathway
NGF Signaling
B Cell Activating Factor Signaling
Activation of IRF by Cytosolic Pattern Recognition Receptors
NER (Nucleotide Excision Repair, Enhanced Pathway)

Figure S3: The canonical pathways enriched from IPA analysis of differentially regulated proteins in optic nerve (glaucoma vs control). Red and green indicate relative increases or decreases in functional enrichment, respectively. The asterisks (*) indicate z-score values of -1.5 to 1.5 and p-values < 0.05 which represented no significant. IPA, ingenuity pathway analysis.
