# Supplementary material for: Early Proteomic Characteristics and Changes in the Optic Nerve Head, Optic Nerve, and Retina in a Rat Model of Ocular Hypertension
Source: Mol Cell Proteomics. 2023 Oct 2;22(11):100654. doi: 10.1016/j.mcpro.2023.100654 (PMC10665672; doi:10.1016/j.mcpro.2023.100654)
Supplement: CWS_Editorial_Certificate [file mmc7.pdf]

Charlesworth Author Services

EDITORIAL CERTIFICATE

This document certifies that the manuscript below was edited for correct English language usage, grammar, punctuation and spelling by qualified native English speaking editors at Charlesworth Author Services.

Paper Title:

Early proteomic characteristics and changes of optic nerve head, optic nerve and retina in rat model of ocular hypertension

Author:

Danting Lin

Date certificate issued:

January 31, 2023

cwauthors.com
